# HPLC-DAD Fingerprints Combined With Multivariate Analysis of Epimedii Folium From Major Producing Areas in Eastern Asia: Effect of Geographical Origin and Species

**DOI:** 10.3389/fphar.2021.761551

**Published:** 2021-11-26

**Authors:** Ben Li, Marta R. M. Lima, Yuhao Nie, Long Xu, Xiang Liu, Hongchao Yuan, Chen Chen, Alberto CP Dias, Xiaoying Zhang

**Affiliations:** ^1^ Chinese-German Joint Laboratory for Natural Product Research, Qinling-Bashan Mountains Bioresources Comprehensive Development C.I.C., College of Biological Science and Engineering, Shaanxi University of Technology, Hanzhong, China; ^2^ Department of Agriculture Nutrition and Food Systems, University of New Hampshire, Durham, NH, United States; ^3^ Centre of Molecular and Environmental Biology (CBMA), Department of Biology, University of Minho, Campus de Gualtar, Braga, Portugal; ^4^ Jinhuifang Traditional Chinese Medicine Technology Co., Ltd, Hanzhong, China; ^5^ Centre of Biological Engineering (CEB), University of Minho, Campus de Gualtar, Braga, Portugal; ^6^ Department of Biomedical Sciences, Ontario Veterinary College, University of Guelph, Guelph, ON, Canada

**Keywords:** Epimedium sp, high performance liquid chromatography (HPLC), principal component analysis (PCA), epimedin C, icariin, baohuoside I

## Abstract

The growth location and plant variety may influence the active components and biological activities of plants used in phytomedicine. In this study, nine sets of different Epimedii Folium, from different representative cultivation locations and Epimedium species, were collected for comparison, using HPLC-DAD combined with multivariate analysis. The objective was to investigate the influence of geographical origin and Epimedium species on the quality of Epimedii Folium, and provide applicable guidance for cultivation and quality control of Epimedii Folium. Several Epimedium spp. sets were used to establish the HPLC-DAD fingerprints and 91 peaks (compounds) were selected for the multivariate analysis. Major compounds were analyzed by HPLC-DAD combined with principal component analysis (PCA). HPLC quantitative analysis of known bioactive compounds was performed. Application of PCA to HPLC data showed that Epimedium samples sharing the same geographical origin or species clustered together, indicating that both species and geographical origin have impacts on the quality of Epimedii Folium. The major bioactive flavonoid compounds, epimedin C, icariin and baohuoside I, were identified and quantified. The concentration of bioactive compounds was significantly influenced both by species and geographical origin. *E. sagittatum* from Sichuan showed the highest content of bioactive compounds. The results showed that both Epimedium species and geographical origin have strong impact into quality of Epimedii Folium. HPLC data combined with multivariate analysis is a suitable approach to inform the selection of cultivation areas and choose Epimedium spp. most suitable for different geographical areas, resulting in improved quality of Epimedii Folium.

## Introduction

Epimedii Folium, “淫羊藿 (Yin Yang Huo)” in Chinese - also known as Herba Epimedii, barrenwort, bishop’s hat, fairy wings, horny goat weed, and rowdy lamb herb - is an important medicinal herb ingredient used in traditional Chinese medicine (TCM) to treat osteoporosis and sexual dysfunction, among other conditions (Ma et al., 2011). Epimedii Folium has been used for more than 2000 years with the major functions of “tonifying kidney Yang, strengthening muscles and bones, dispelling wind and dampness” (Chen et al., 2015b). Epimedium sp. improved osteoporosis condition and strengthening bones in human studies (Indran et al., 2016), and has been used to treat sexual dysfunction (Shindel et al., 2010) and cardiovascular diseases (Li et al., 2015b). Nowadays, Chinese Pharmacopeia accepts four Epimedium species as a source of Epimedii Folium, including *Epimedium brevicornum* Maxim*, Epimedium sagittatum* (Siebold and Zucc.) Maxim*, Epimedium pubescens* Maxim*,* and *Epimedium koreanum* Nakai. Its dried leaves have spicy and sweet tastes, and have been used for further dosage preparations (Chinese Pharmacopoeia, 2020).

Many active compounds, including epimedin A, epimedin B, epimedin C, icariin and baohuoside I, have been identified from Epimedium (Wu et al., 2012). Among them, the prenylflavonoids flavonoids icariin, epimedin C and baohuoside I, are considered as the major bioactive components and used as marker compounds for quality control (Zhao et al., 2010). Icariin, a flavonol glycoside obtained from the aerial part of the plant (Indran et al., 2016), could enhance the osteogenic effect of bone morphogenetic protein 2 (BMP2) which induces osteoblast differentiation and stimulate bone or cartilage formation and cyclic adenosine monophosphate (cAMP) signaling pathway which regulates osteogenic differentiation and mineralization (Chen et al., 2019). Additionally, icariin has been reported to have anti-tumorigenic activity. Icariin significantly inhibited the proliferation of several cancer cells, like ovarian cancer cells (Li et al., 2015a), medulloblastoma cells (Sun et al., 2016), and human neural cells (Yang et al., 2016).

*Epimedium brevicornum* Maxim is widely distributed in northwest China, including Gansu, Shaanxi, Ningxia and He’nan provinces, whilst *Epimedium pubescens* Maxim grows in the south provinces of Sichuan, Guizhou and Anhui (Guo and Xiao, 2003). These two species have been regarded having higher quality with consistent higher levels of major active components (He et al., 2019). Quality of commercial Epimedii Folium is mainly controlled by its icariin content, with the minimal content of 0.5% (g/g DW) in dried products, according to Chinese Pharmacopoeia (Chinese Pharmacopoeia, 2015). However, icariin contents of Epimedium on the markets remain uneven, even undetectable in some batches of commercial Epimedium, possibly due to the regional and varietal differences. According to a survey performed in 2014, the ranges of icariin contents in 104 batches from different species were 0.01–0.17% (g/g DW) and all of them were substandard (Ma et al., 2014). Other studies support such observation (Pei et al., 2007; Polat and Coskun, 2016).

This study aimed to investigate the influence of the cultivation location (province) and Epimedium species on the phytocomposition and quality of Epimedii Folium, namely the major relevant bioactive components, using HPLC-DAD and multivariate statistical analysis, since these issues are highly relevant for cultivation and quality control of Epimedii Folium.

## Materials and Methods

### Chemicals

HPLC-grade ethanol, acetonitrile and formic acid were purchased from Chron chemicals (Chengdu, Sichuan, China), Damao chemical (Tianjin, China) and Kermel Chemical (Tianjin, China), respectively. Ultrapure water with a resistivity of 18 M^Ω^.cm at 25°C was generated with Microporous system (Ulu pure, Xian, Shaanxi, China). The analytical standards were purchased from Desite (Chengdu, Sichuan, China): Epimedin C (purity >98%), icariin (purity >98%) and Baohuoside I (purity >99%).

### Collection and Preparation of Epimedium sp. Samples

**FIGURE 1:**
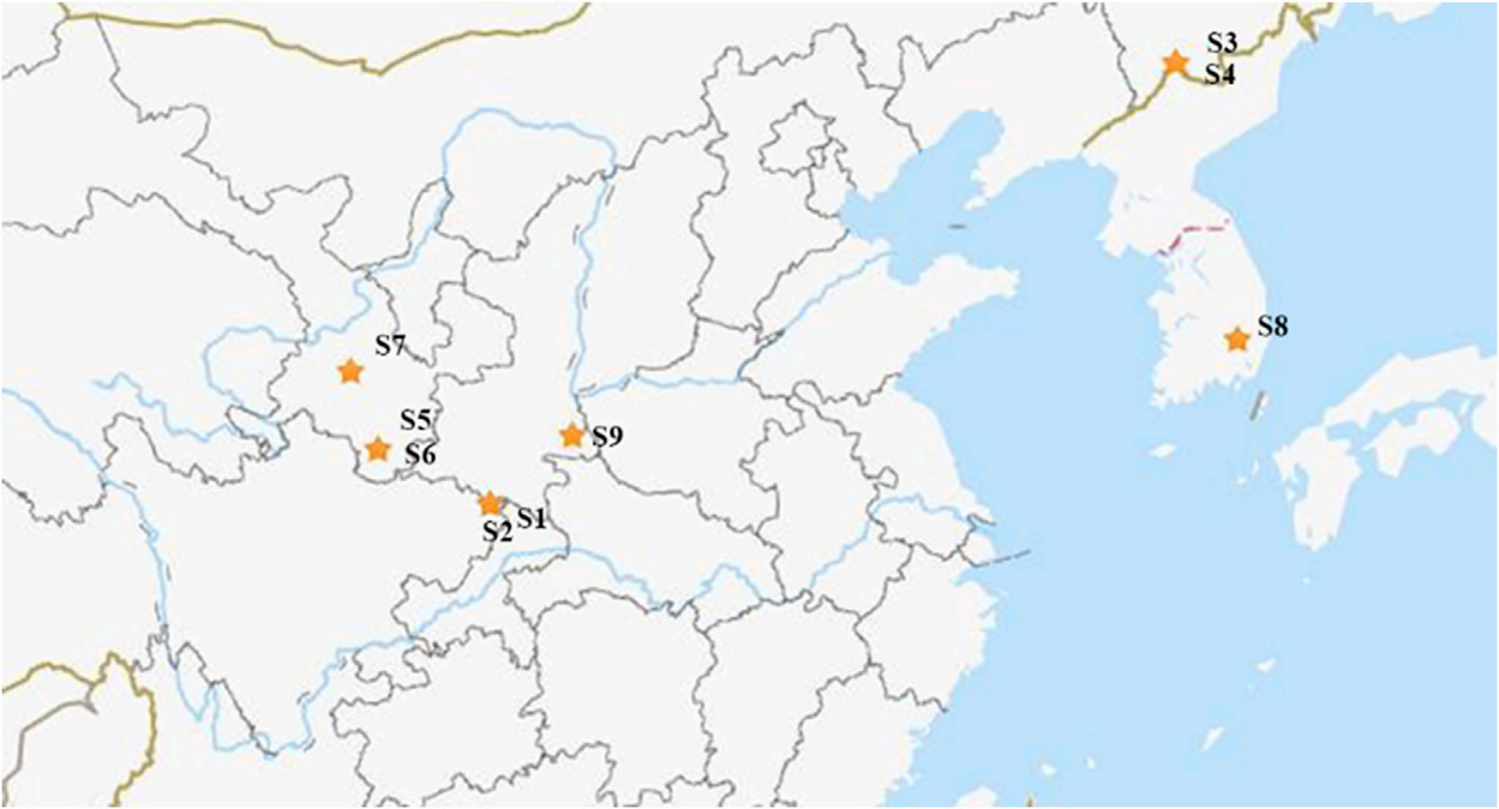
Geographical distribution of Epimedium samples collected in this study.

Leaves of *E. pubescens* and *E. sagittatum* were collected at a cultivation field located at Wanyuan (Sichuan) (S1 and S2 samples, Table 1). Other Epimedium samples were purchased directly from local certified TCM markets, with a valid and clear certificate of origin, provided by Chinese official regulators (State Administration for Market Regulation). All the samples were further verified and confirmed by experts and voucher specimens were deposited in the herbarium collection of College of Biological Science and Engineering, Shaanxi University of Technology, Hanzhong, China. The species and respective origin are listed in Table 1 and geographical locations are shown in Figure 1. From each location/species, five independent samples were obtained based on batch leaves from individual plants, to account for normal *in vivo* variability.

**TABLE 1:** Sources and species of Epimedii Folium samples.

Sample	Origin	Species
S1	Wanyuan, Sichuan	*E. pubescens* Maxim
S2	Wanyuan, Sichuan	*E. sagittatum* (Siebold and Zucc.) Maxim
S3	Linjiang, Jilin	*E. pubescens* Maxim
S4	Linjiang, Jilin	*E. koreanum* Nakai
S5	Longnan, Gansu	*E. pubescens* Maxim
S6	Longnan, Gansu	*E. brevicornum* Maxim
S7	Weiyuan, Gansu	*E. brevicornum* Maxim
S8	Daqiu, South Korea	*E. koreanum* Nakai
S9	Shangluo, Shaanxi	*E. brevicornum* Maxim

The leaves were dried by lyophilization to constant weight, milled into powder, and stored in the dark at room temperature until use. Aliquots (0.2 g) of powder samples of Epimedium were weighed and added to 8 ml of 70% aqueous ethanol. Extraction was done using sonication for 2 min × 30 min. After this, solutions were centrifuged at 13,500 *g* for 5 min, the supernatant was filtered through 0.22 μm Nylon six microporous filter membrane, and the filtrate was collected in amber borosilicate glass vials for HPLC-DAD analysis.

### HPLC-DAD Analysis

Samples were injected into a liquid chromatograph system UltiMate 3000 (Thermo, Waltham, MA, United States). Chromatographic separations were achieved using gradient elution on an Inertsil ODS-3 column (150 mm × 4.6 mm, 4 μm). Mobile phase A was acetonitrile containing 0.1% formic acid and mobile phase B was ultrapure water containing 0.1% formic acid. The gradient elution program was set as follows: 80% (B) for 0–3 min, 80%–70% (B) for 3–15 min, 70% (B) for 10–15 min, 70%–10% (B) for 15–30 min, 10% (B) for 30–35 min, 10%–80% (B) for 35–40 min. The flow rate was 0.75 ml/min. The column was maintained at 30°C and the sample injection volume was 10 μL. The detection wavelength was recorded between 230 and 600 nm, and chromatograms were recorded at 274 nm. Quantification of epimedin C, icariin and baohuoside I was made at 274 nm based on the external standard method using standard curves of commercial pure compounds.

The HPLC chromatograms were exported as txt ASCII files and the chromatographic fingerprint process was drew using Origin Lab Pro version 9.4 (Origin Lab software, Northampton, MA, United States).

### Statistical Analysis

A total of 91 peaks in the HPLC chromatograms of the nine Epimedium sets (45 independent samples in total) were selected for multivariate statistical analysis. Peaks were manually aligned based on their retention time and UV spectra, to assure common identity, and named 1 to 91. Peak areas (274 nm) were corrected by the amount of biomass extracted. The resulting table was imported into GraphPad Prism version 9.1.1 for Windows (GraphPad Software, San Diego, CA, United States, www.graphpad.com). Data was standardized prior to principal component analysis (PCA). The R-statistical software version 4.1.0 (R Core Team, 2021), ggplot2 version 3.3.5 (Wickham, 2016), and ggrepel version 0.9.1 (https://cran.r-project.org/web/packages/ggrepel/index.html) packages were used to display the corresponding plots. The amounts of epimedin C, icariin and baohuoside I from the different Epimedium species cultivated in different regions were plotted and compared in GraphPad Prism using one-way ANOVA followed Tuckey’s test or t-test, to compare three or two groups, respectively. One outlier of *E. sagittatum*, one of *E. pubescens* from Sichuan, and two outliers of *E. brevicornum* from Weiyuan Gansu were removed prior to comparison. Data normality was assessed using the Kolmogorov-Smirnov test. Statistical significance was considered at *p* < 0.05. All matrices were also imported into the SIMCA14.0 software (Umetrics, Umea, Västerbotten, Sweden). The obtained quantification data were scaled with unit variance scaling, and sample subgroups (*E. koreanum* and Sichuan) were subjected to PCA.

## Results and Discussion

### Epimedii Folium HPLC-DAD Analysis

The chemical quality of plants is influenced by both biotic and abiotic environmental factors and known to exhibit extensive geographic variation (Chen et al., 2013). Epimedium is native to China with wide distribution in He’nan, Shanxi, Shaanxi, Gansu, and Ningxia Provinces (although Epimedium spp. can be found in other regions of East Asia such as *E. koreanum* in Japan and North Korea), and has abundant pharmacological functions (Xu et al., 2013; Li et al., 2018). However, safe and effective use of Epimedium has been limited by variation of Epimedium quality, and identification of plant location and variation (Han et al., 2012).

**FIGURE 2:**
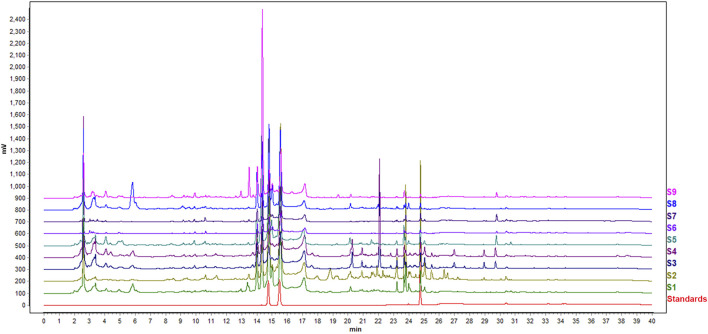
HPLC fingerprints of nine sets of Epimedium samples studied and standards (epimedin C, icariin and baohuoside I, from left to right).

Typical chromatograms resulting from the HPLC analysis of nine sets of Epimedium from different geographical origin and species are shown in Figure 2. There were good chromatogram resolutions in the fingerprint of all Epimedium samples, namely for the standards epimedin C, icariin and baohuoside I, considered quality marker compounds for Epimedii Folium, and the other major compounds found. Differences between the different Epimedium samples could not be easily detected in the chromatograms by simple visual inspection. Hence, HPLC data was subjected to PCA, with the purpose of uncovering an effect related to geographical origin and/or species on the quality of Epimedium.

### PCA Analysis

The use of Multivariate Analysis, like Principal Component Analysis (PCA), is nowadays commonly used for better understanding metabolite diversity, namely of phenolics, and link it with adulterations (Windarsih et al., 2019), biotic stress (Lima et al., 2010), and different geographical and species variation (Chen et al., 2015a).

**FIGURE 3:**
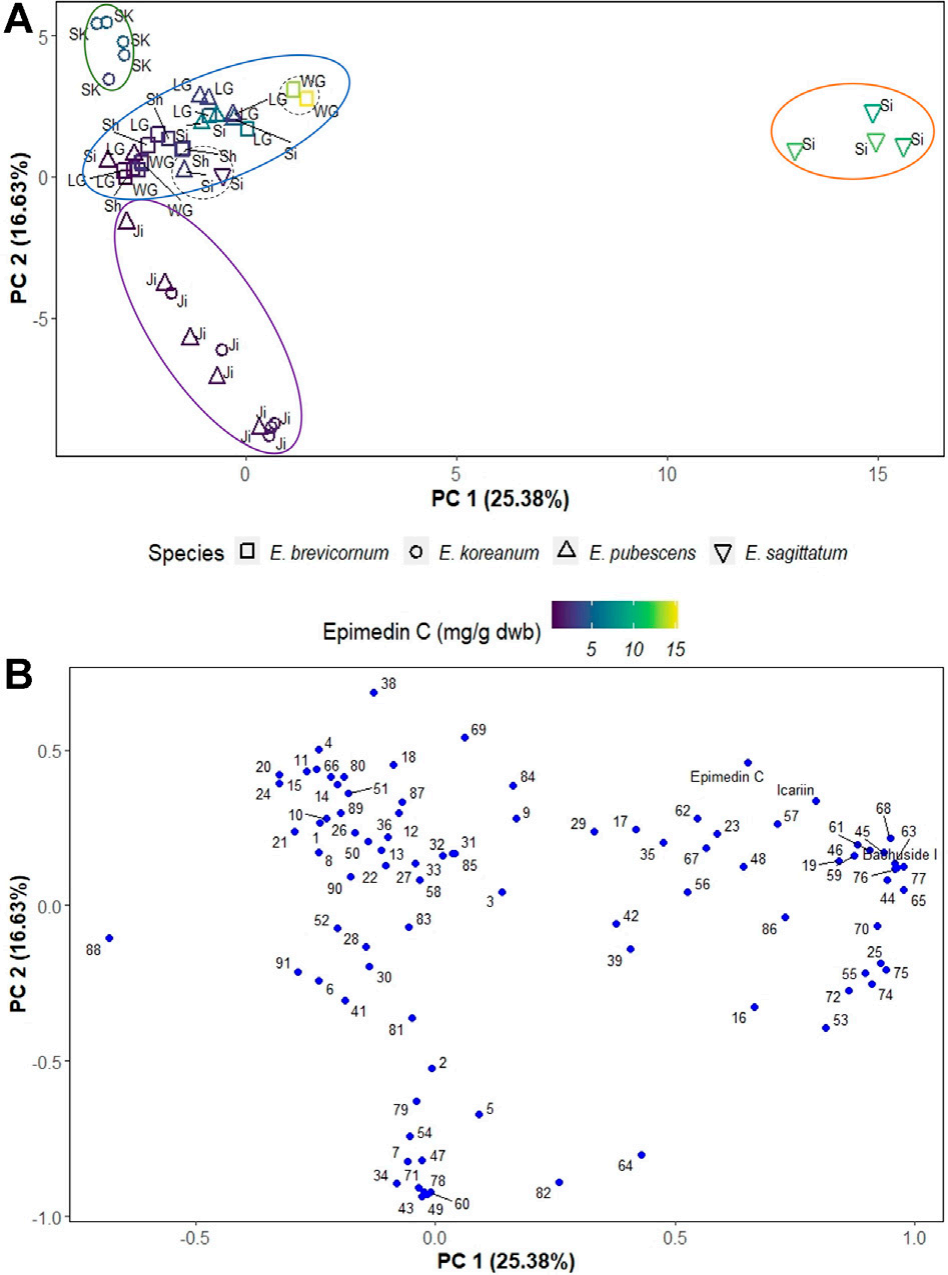
PCA scores plot **(A)** and loadings plot **(B)** of Epimedium samples. In the scores plot, Epimedium species are represented by different shapes, and each data point is colored according to its amount of epimedin C and labeled with its geographical origin (SK—South Korea, LG—Longnan Gansu, WG—Weiyuan Gansu, Ji—Jilin, Si—Sichuan, Sh—Shaanxi). Ellipses indicate sample groups based on species or geographical origin; dashed-line circles indicate outliers (two *E. brevicornum* outliers from Weiyuan Gansu, one E. sagittatum outlier and another E. pubescens both from Sichuan). The loadings plot **(B)** shows the compounds responsible for the group separation; for clarity, the peak numbers corresponding to epimedin C, icariin and baohuoside I were substituted by their name.

In this work, PCA was used to investigate how different species and geographical origin are relevant (or not) for differences and quality of Epimedii Folium. The best discriminating principal components (PCs), PC1 and PC2, cumulatively accounted to the explanation of 42.01% of the total variance in the data. The PC1 and PC2 scores scatter plot (Figure 3A) clearly shows the separation of *E. sagittatum* species from the other Epimedium species along PC1, with *E. sagittatum* samples clustering towards higher positive values of PC1 (orange ellipse in Figure 3A), and all other species grouping towards lower and negative values of PC1.

Additionally, PC2 clearly separates Epimedium samples based on geographical origin. Samples from South Korea clustered towards the highest values of PC2 (green ellipse in Figure 3A), the samples from central China provinces (Gansu, Sichuan, and Shaanxi) clustered at lower values of PC2 (blue ellipse in Figure 3A), and the samples from the Jilin province, in northeast China, clustered towards negative values of PC2 (purple ellipse in Figure 3A).

The PC1 and PC2 loadings plot (Figure 3B) shows the compounds contributing to the separation of Epimedium samples into different groups. The pharmacologically active compounds epimedin C, icariin and baohuoside I, were among the compounds that most contribute to sample separation, because they are associated with higher PC1 values and positive PC2 values. To further confirm the importance of these bioactive compounds in separating Epimedium samples, the data points in the scores scatter plot were colored according to a gradient based on epimedin C concentration (Figure 3A). The *E. sagittatum* samples contained higher amounts of Epidemium C (orange ellipse), followed by the *E. koreanum* samples from South Korea with medium-high amounts (green ellipse), then the *E. pubescens* and *E. brevicornum* samples from central China with medium-low concentration of epimedin C (blue ellipse), and finally the *E. koreanum* and *E. pubescens* samples from the Jilin province containing the lowest amounts of epimedin C (purple ellipse).

**FIGURE 4:**
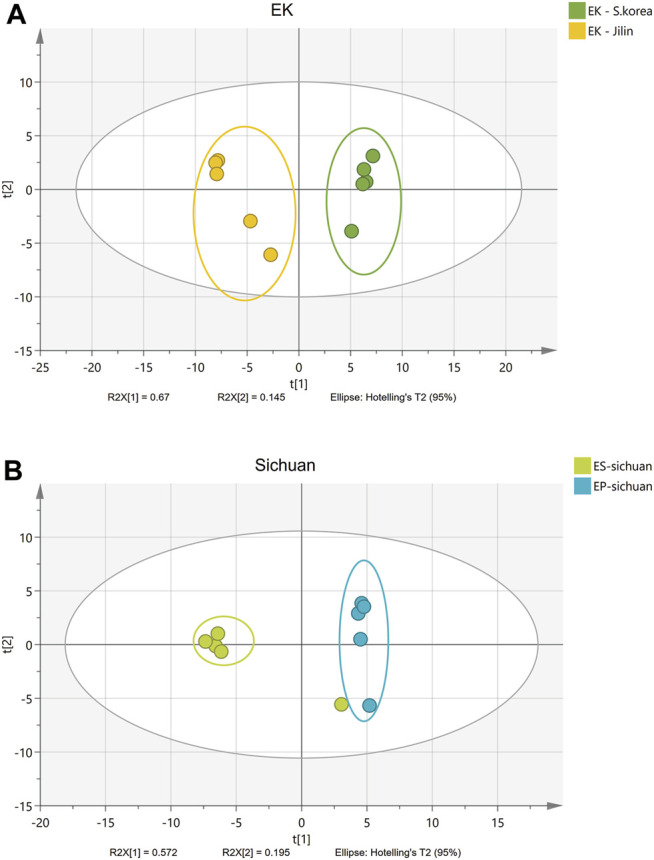
PCA scores plot **(A)** for same Epimedium species (*E. koreanum*, EK) collected in different locations (Daqiu, South Korea and Jilin, China)—A; and PCA scores plot **(B)** for different species (*E. sagittatum*, EK, and *E. pubescens*, EP) cultivated in the same field (Wanyuan, Sichuan).

Some isolated studies indicated that the compounds of Epimedium from neighboring locations were similar (Huang et al., 2007; Xu et al., 2013; Xu et al., 2017). This study included samples from a wide geographical area and from different species (Table 1; Figure 1), as a way to offer a more comprehensive view of how location and species may affect biomass quality. In fact, using PCA analysis, it was easy to distinguish Epimedii Folium from the same species but from different provenience (Figure 4A), and from different species grown in the same cultivated field, under the same abiotic and biotic stressors (Figure 4B). It is clear that, both Epimedium species and provenience have strong impact on the phenolic contents and quality of Epimedii Folium.

### Content Differences of Bioactive Components in Epimedium Sets

**FIGURE 5:**
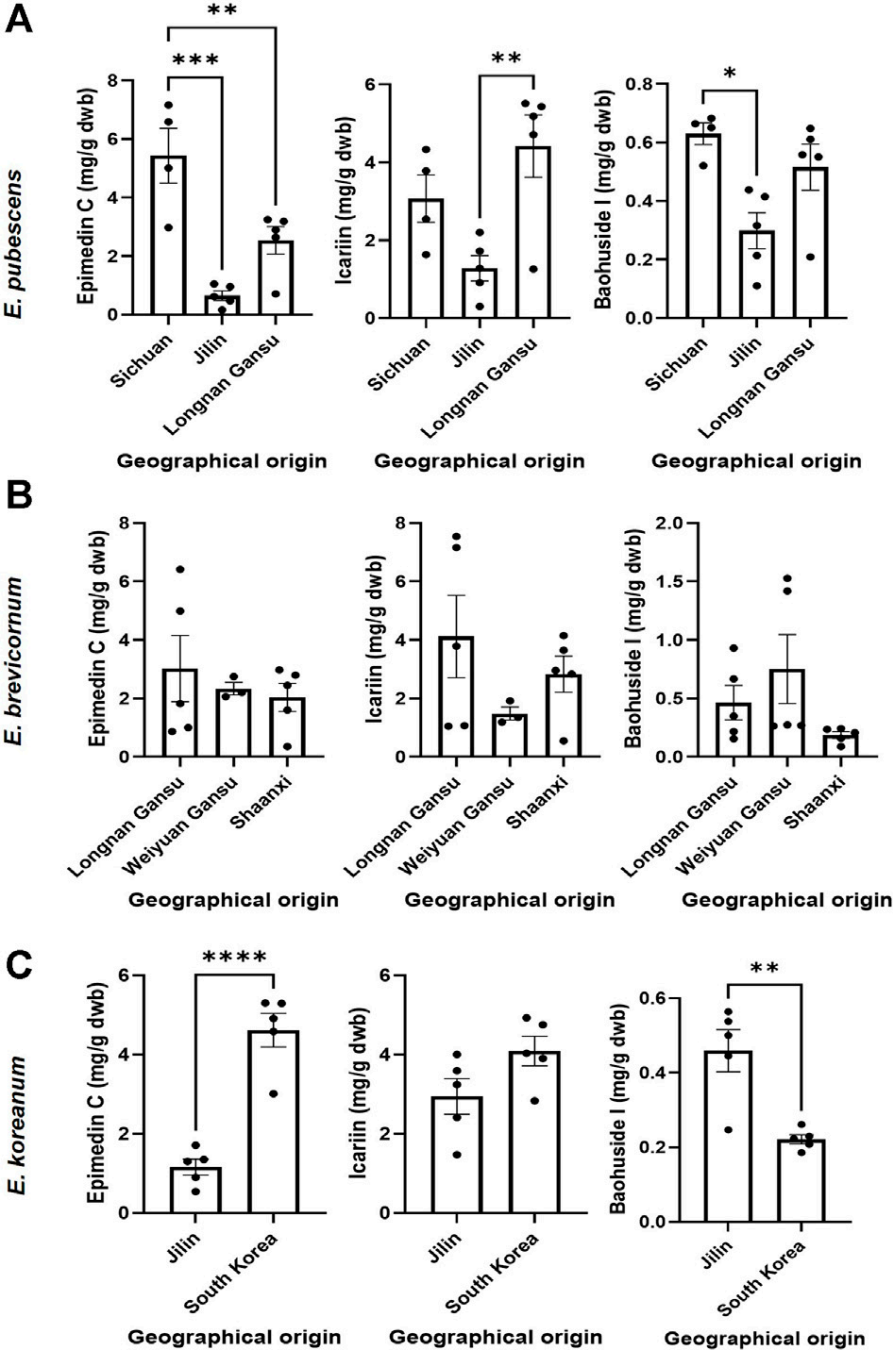
Amount (mg/g dry weight biomass) of epimedin C, icariin and baohuoside I in *E. pubescens* **(A)**, *E. brevicornum* **(B)** and *E. koreanum* **(C)** cultivated in different geographical locations. Bars represent average ±SEM; dots represent individual data points. Statistically significant differences are represented by asterisks (*p < 0.05, **p < 0.01, ***p < 0.001, ****p < 0.0001).

**FIGURE 6:**
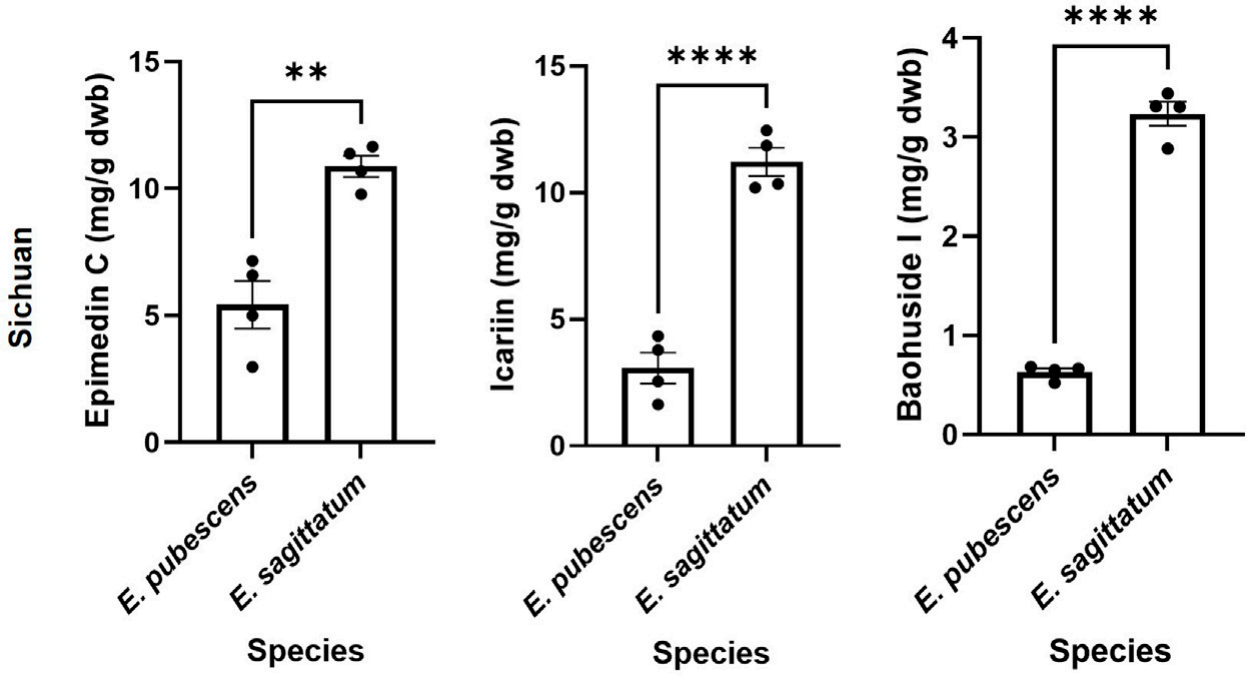
Amount (mg/g dry weight biomass) of epimedin C, icariin and baohuoside I of different plant species cultivated in same place (Wanyuan, Sichuan). Bars represent average ±SEM; dots represent individual data points. Statistically significant differences are represented by asterisks (**p < 0.01, ****p < 0.0001).

The relevant bioactive compounds to Epimedii Folium (or Herba Epimedii) used in TCM, epimedin C, icariin, and baohuoside I, were quantified in the samples studied (Figures 5, 6). *E. sagittatum* contained the highest amounts of epimedin C (10.88 ± 0.83 mg/g dwb), icariin (11.21 ± 1.12 mg/g dwb), and baohuoside I (3.23 ± 0.24 mg/g dwb). The bioactive amounts in the other Epimedium species were lower and varied according to geographical origin (Figure 5). For *E. pubescens* (Figure 5A), average epimedin C concentration was significantly higher when cultivated in Sichuan (5.43 ± 1.87 mg/g dwb) compared to *E. pubescens* samples from Longnan Gansu (2.54 ± 1.05 mg/g dwb) and Jilin (0.65 ± 0.36 mg/g dwb). Average *E. pubescens* icariin concentration was significantly higher in samples from Longnan Gansu (4.42 ± 1.79 mg/g dwb) when compared to samples from Jilin (1.28 ± 0.73 mg/g dwb), but not significantly different from Sichuan samples (3.07 ± 1.22 mg/g dwb). Average *E. pubescens* baohuoside I concentration was significantly higher in samples from Sichuan (0.63 ± 0.07 mg/g dwb) when compared to samples from Jilin (0.30 ± 0.14 mg/g dwb), but not significantly different from Longnan Gansu samples (0.51 ± 0.17 mg/g dwb). It is noteworthy to mention that some of the species indicated by producers as having the highest contents in bioactive contents might be different. As an example, *E. pubescens* was considered to have the highest contents in bioactives (He et al., 2019). Nevertheless, for *E. sagittatum* and *E. pubescens* plants cultivated in the same field location, under the same abiotic and biotic environment, the bioactive contents (icariin, epimedin C, baohuoside I) were significantly higher for *E. sagittatum* (Figure 6).

For *E. brevicornum* (Figure 5B) the amount of the identified bioactive components varied, on average, between 2.04 and 3.02 mg/g dwb for epimidium C, between 1.49 and 4.12 mg/g dwb for icariin, and between 0.19 and 0.75 mg/g dwb for baohuoside I. No significant differences were detected among the different geographical regions, likely because all the *E. brevicornum* samples analyzed in this study were cultivated in regions exclusively located to central China. For *E. koreanum* (Figure 5C), average epimedin C concentration was significantly higher when cultivated in South Korea (4.62 ± 0.95 mg/g dwb) compared to *E. koreanum* samples from Jilin (1.16 ± 0.45 mg/g dwb). Average E. koreanum icariin content was not significantly different in samples from South Korea (4.10 ± 0.83 mg/g dwb) compared to samples from Jilin (2.94 ± 1.01 mg/g dwb). Average *E. koreanum* baohuoside I concentration was significantly higher in samples from Jilin (0.46 ± 0.13 mg/g dwb) compared to samples from South Korea (0.22 ± 0.03 mg/g dwb).

In our current study, the contents of icariin in *E. sagittatum* from Sichuan and *E. brevicornum* from Gansu Wanyuan were above standard according to the 2015 Chinese Pharmacopoeia (Chinese Pharmacopoeia, 2015). However, in the current Chinese Pharmacopoeia released in 2020 (Chinese Pharmacopoeia, 2020), the standard for Epimedium quality control has been changed to the analysis of the icariin content and calculation of the total amount of Epimedium A, B, C and icariin based on the correction factor, that means, *E. pubescens* from Jilin and *E. brevicornum* from Shaanxi were below standard. There is clear difference between the two editions of the pharmacopoeia standards, despite the content of icariin has been the major quality consideration in both editions. It is noteworthy that, according to the results of the recent studies, the contents of icariin in Epimedium were easily affected by external factors (Chen et al., 2015b; Deng et al., 2018; Li et al., 2020). Therefore, finding the variation patterns of the content of various components in Epimedium herbs of different origins and varieties and screening the appropriate content determination index are the keys to solve the current Epimedium quality control issue.

Overall, both species variant and geographical location influence the contents of bioactive components in Epimedii Folium, and so the pharmacology quality of the biomass (Wei et al., 2017; Yuan et al., 2017). Therefore, it is necessary to explore the geo-herbalism of Herba epimedii by the characteristic component variation and chromatographic fingerprint among different sets. *E. koreanum* belongs to large-flowered taxa and *E. pubescens, E. sagittatum* and *E. brevicornum* all belong to small-flowered taxa (Xu and He, 2005). However, the icariin content of *E. sagittatum* was significantly higher compared with *E. pubescens* cultivated in the same region (Figure 6). *E. sagittatum* also showed relatively independent from *E. pubescens*, *E. koreanum* and *E. brevicornum* through PCA scores plot compared with *E. wushanense* (Xie et al., 2010), which proved that Epimedium species variation is a factor in the interspecific differences, and indicated that the differences between different species of Epimedium should be explored.

As a conclusion, the use of HPLC-DAD combined with multivariate analysis (PCA) is an effective methodology to discriminate different Epimedii Folium samples from different epimedium species and geographical origins. Our results provide applicable guidance to the geographical location and plant species selection of GAP (Good Agricultural Practices) production for Epimedii Folium. Both species and geographical location variations have impacts on the quality and composition of Epimedii Folium. However, the components of herbal products are diverse and complex, and their pharmacological activities are always affected by unique component constituents as well as their combinations, instead of a single component (Zhang et al., 2013). Therefore, associations between the variation of plant species and geographical locations with pharmacological activity of Epimedii Folium need to be further explored for providing better evaluation criteria for geo-herbalism of Epimedii Folium.

## Data Availability

The original contributions presented in the study are included in the article/Supplementary Material, further inquiries can be directed to the corresponding authors.
